# Casein Kinase 1 Alpha Regulates Chromosome Congression and Separation during Mouse Oocyte Meiotic Maturation and Early Embryo Development

**DOI:** 10.1371/journal.pone.0063173

**Published:** 2013-05-15

**Authors:** Lu Wang, Angeleem Lu, Hong-Xia Zhou, Ran Sun, Jie Zhao, Cheng-Jie Zhou, Jiang-Peng Shen, Sha-Na Wu, Cheng-Guang Liang

**Affiliations:** The Key Laboratory of National Education Ministry for Mammalian Reproductive Biology and Biotechnology, Inner Mongolia University, Hohhot, Inner Mongolia, People's Republic of China; Institute of Zoology, Chinese Academy of Sciences, China

## Abstract

Casein kinase I alpha (CK1α) is a member of serine/threonine protein kinase, generally present in all eukaryotes. In mammals, CK1α regulates the transition from interphase to metaphase in mitosis. However, little is known about its role in meiosis. Here we examined *Ck1α* mRNA and protein expression, as well as its subcellular localization in mouse oocytes from germinal vesicle to the late 1-cell stage. Our results showed that the expression level of CK1α was increased in metaphase. Immunostaining results showed that CK1α colocalized with condensed chromosomes during oocyte meiotic maturation and early embryo development. We used the loss-of-function approach by employing CK1α specific morpholino injection to block the function of CK1α. This functional blocking leads to failure of polar body 1 (PB1) extrusion, chromosome misalignment and MII plate incrassation. We further found that D4476, a specific and efficient CK1 inhibitor, decreased the rate of PB1 extrusion. Moreover, D4476 resulted in giant polar body extrusion, oocyte pro-MI arrest, chromosome congression failure and impairment of embryo developmental potential. In addition, we employed pyrvinium pamoate (PP), an allosteric activator of CK1α, to enhance CK1α activity in oocytes. Supplementation of PP induced oocyte meiotic maturation failure, severe congression abnormalities and misalignment of chromosomes. Taken together, our study for the first time demonstrates that CK1α is required for chromosome alignment and segregation during oocyte meiotic maturation and early embryo development.

## Introduction

In mammals, oocyte maturation involves the process of meiosis. Meiosis is characterized by two rounds of chromosome segregation but only one round of DNA replication. This is the foundation of propagation and inheritance in sexual reproduction. In oogenesis, homologous chromosomes separate during anaphase of meiosis I and extrude a tiny non-developmentally competent cell, the first polar body (PB1), which marks the oocyte's nuclear maturation. Fertilization triggers the second meiosis, in which sister chromatids segregate, resulting in second polar body (PB2) extrusion. Errors in chromosome separation result in aneuploidy, associated with oocyte maturation failure and consequences for aneuploidy or dysplasia in the embryo [1]. The mechanism of regulating chromosome dynamics is highly conservative and precise.

A variety of proteins regulate chromosome alignment and separation through phosphorylation, dephosphorylation, ubiquitination pathways. In this process, spindle assembly checkpoint (SAC) mechanisms ensure that all chromosomes are properly attached to the spindle before onset of anaphase [2]. Any SAC molecular deficiency results in the oocyte's metaphase arrest. In addition, a large number of protein phosphorylation/dephosphorylation events take place which ensures that mRNA and proteins are adequately synthesized in oocytes and accurately regulate chromosome dynamics, spindle assembly/disassembly, chromosome distribution and oocyte arrest at the MII stage until fertilization takes place. Thus, protein kinase-mediated regulatory pathways are essential to achieve high quality-matured oocytes, and the ability to support subsequent fertilization and embryo development.

In recent years, a large number of protein kinases have been reported to be involved in oocyte maturation. Some candidates have been widely investigated for their functions, *e.g.*, Aurora kinase A (AURKA) regulates MTOC number and spindle length [3], Aurora Kinase B modulates chromosome alignment [4], Extracellular Signal-regulated Kinase 3 (ERK3) stabilizes the spindle and plays a key role in the metaphase- anaphase transition [5]. However, little is known about the function of the casein kinase 1 family in meiosis.

The casein kinase 1 family is a family of serine/threonine protein kinases, commonly existing in all eukaryotes [6]. A number of studies have shown that casein kinase 1 is involved in Wnt signaling pathways [7]. Their functions include roles in circadian rhythms [8], cellular transformation and mammary carcinogenesis [9], nucleo-cytoplasmic shuttling of transcription factors [10], DNA repair [11], and mRNA metabolism [12]. Until now, seven family members were identified, which are encoded by different genes, including alpha, beta 1, gamma 1, gamma 2, gamma 3, delta, and epsilon. They are highly homologous with more than 50% identical kinase domains [13]–[15]. Previous studies revealed that casein kinase 1 delta/epsilon isoforms Hhp1 and Hhp2 are required for chromosomes segregation by removal of Rec8 during first meiosis in the fission yeast [16]. It was also shown to function as cohesin kinase to facilitate cleavage during the meiosis I [17]. Also, CK1α has been shown to play a role in the regulation of chromosome segregation during mitosis in mammalian cells [18]. In addition, previous studies showed that CK1α exists and is activated in mouse oocytes and that it regulates the interphase-mitosis transition during the first mitotic cell cycle in embryos [19]. Recently, we investigated CK1α in somatic cell nuclear transfer (SCNT) embryos and found that CK1α was significantly decreased in SCNT constructs, accompanied by chromosomes misalignment in SCNT embryos [20]. However, the detailed mechanism of how CK1α acts on oocyte meiosis and early embryo development is still unknown.

In the present study, we investigated expression, localization and function of CK1α during mouse oocyte meiotic maturation and early embryo development. We also studied CK1α's functions on chromosome alignment by functional depletion, pharmaceutical inhibition and activation approaches.

## Materials and Methods

### Ethics statement

All studies adhered to procedures consistent with the National Research Council Guide for the Care and Use of Laboratory Animals and were approved by the Institutional Animal Care and Use Committee at Inner Mongolia University.

### Oocyte collection

Adult female (B6D2) F1 mice (8–12 weeks of age) were used for the sample collections. All chemicals and media were purchased from Sigma Aldrich Company (St. Louis, MO) unless stated otherwise. For *in vivo* MII stage oocyte collection, mice were superovulated with 10 IU of pregnant mare serum gonadotropin (PMSG, SanSheng, Ningbo, China) followed by 10 IU of human chorionic gonadotropin (hCG, SanSheng) 48 hours later. The MII oocytes with cumulus mass were released from the oviduct ampullae at 14–16 hours of hCG injection. Cumulus cells were dispersed by 0.3 mg/mL hyaluronidase in HEPES-M2 medium. Oocytes were cultured in Chatot-Ziomet-Bavister (CZB) medium for 30 minutes of recovery. GV oocytes were collected by puncturing the follicles of ovaries at 48 hours of PMSG injection. Cumulus cells were removed by gentle pipeting.

### IVF and embryo culture

Spermatozoa were collected from the cauda epididymis of adult male (B6D2) F1 mice at 10–14 weeks of age. The sperm suspension was capacitated for 2 hours in 200 µL of T6 medium supplemented with 4 mg/ml BSA. MII oocytes were incubated with spermatozoa for 6 hours. Sperm concentration for fertilization was 1×10^6^/mL. The zygotes were cultured in CZB medium without glucose under a humidified atmosphere of 5% CO_2_ at 37°C for the first two days and then transferred to CZB medium supplemented with 5.5 mmol/L glucose when embryos reached the 4-cell stage.

### Real-time quantitative PCR analysis

Analysis of *Ck1α* mRNA was measured by real-time quantitative PCR and the ΔΔCT method. Total RNA was extracted from 50 oocytes/zygotes using a PicoPure RNA Isolation Kit (Applied Biosystems, Carlsbad, CA) following the manufacturer's instructions. Oocyte cDNA was synthesized using PrimeScript RT reagent Kit (TaKaRa) following the manufacturer's instructions. Primers for mouse *Ck1α* was forward, TAC GCC AGC ATC AAT GCA CA; reverse, CAA CAC CTC AAC AGG AGT GGA CA. Primers for mouse *Gapdh* was forward, TGT GTC CGT CGT GGA TCT GA; reverse, TTG CTG TTG AAG TCG CAG GAG. SYBR® Premix Ex Taq™ II (TaKaRa) was used for cDNA amplification. The comparative Ct method was used for data analysis and *Gapdh* was used for internal control.

### Western blot

Oocytes were collected in Laemmli Sample Buffer (Bio-Rad Hercules, CA) containing 1/2000 (v/v) Protease Inhibitor Cocktail and 1/20 (v/v) β-mercaptoethanol (Amresco, Solon, OH). All the other western blot procedures were conducted as previously reported [21]. We used rabbit anti-casein kinase 1 alpha (1∶1000, Abcam, Cambridge, MA) and rabbit anti-beta tubulin (1∶500, Abcam) as primary antibodies. Perox-AffiniPure Dnk Anti-Rabbit IgG (H+L) (Jackson ImmunoResearch, West Grove, PA) diluted in 0.5% non-fat milk were used as second antibody. Blots were processed by X-ray exposure to visualize CK1α (39 kDa) and TUBB (53 kDa) bands.

### Immunofluorescence and confocal microscopy

Oocytes and embryos were exposed to acidic tyrode solution (pH 2.5) for a few seconds to remove the zona pellucida followed by three times of washing in M2 medium. Oocytes were then fixed in 4% paraformaldehyde (Electron Microscopy Sciences, Hatfield, PA) in PBS at room temperature for 50 minutes, followed by permeabilization in PBS containing 0.5% Triton X-100 for 2 hours. Sample blocking was conducted with 1% bovine serum albumin (BSA, Amresco) in PBS containing 1/1,000 Tween-20 (Amresco) and 1/10,000 Triton X-100. After blocking, samples are incubated with primary antibodies at 4°C overnight. For primary antibodies, we used rabbit anti-Casein Kinase 1 alpha antibody (1∶100, Abcam) and mouse anti-beta Tubulin (1∶2000, Abcam). For secondary antibodies, we used FITC conjugated donkey anti-mouse (1∶100, Jackson Immuno Research Laboratories) or Dylight-488 conjugated donkey anti-rabbit IgG (H+L) (1∶1000, Jackson Immuno Research Laboratories). DNA was stained with 5 µg/ml Propidium iodide (PI).

After staining, samples were mounted on glass slides using vectashield (Vector Labs, Burlingame, CA) mounting medium and examined with a confocal laser scanning microscope (Nikon, A1R, Japan). Images of optical sections were captured at 1 µm intervals. Images were analyzed with the NIS-Element AR3.0 software. MII plate thickness was measured by drawing two lines at the edges of the red region (PI staining) and perpendicular to the spindle axis defined by the green region (TUBB staining). The distance between these two lines was used as the MII plate thickness. Each experiment was repeated at least three times.

### Morpholino injection

For *Ck1α* knock down in mouse oocytes, *Ck1α* morpholino (MO) 5′- TGC TCG CCA TCC TGA GAC GCG AAG A-3′ (Gene Tools, Philomath, OR) was diluted with water to give a stock concentration of 2 mmol/L. Fully grown GV oocytes were microinjected with 5 pL solution in M2 medium containing 2.5 µmol/L morpholinos with pico-injector (Harvard Apparatus, Holliston, MA) under an inverted microscope (Olympus IX71, Japan). Oocytes were incubated in CZB medium containing milrinone for 20 hours, then washed three times in milrinone-free CZB medium and cultured for meiosis resumption and maturation. Oocytes were cultured for 12 hours for MII stage sample collection. The control group was injected with MO standard control 5′-CCT CTT ACC TCA GTT ACA ATT TAT A-3′.

### Drug treatment

D4476 (4-[4-(2,3-dihydro-benzo[1,4]dioxin-6-yl)-5-pyridin-2-yl-1H-imidazol-2-yl] benzamide) stock solution was diluted to 50 mmol/L in dimethyl sulfoxide (DMSO) and further diluted in CZB medium to a final concentration of 50 µmol/L. Pyrvinium pamoate (PP) stock solutions were prepared at 10 mmol/L in DMSO and further diluted in CZB medium to a final concentration of 3 nmol/L. Oocytes were exposed to D4476 or PP containing CZB medium for 12 hours. Samples were collected for subsequent experimental procedures.

### PB1 size measurement

PB1 sizes were measured by CellSens Standard software (Olympus, Japan). Single oocytes were rotated to ensure that the polar body was upward above the oocyte for acquisition of length and width. Then the oocytes were rotated 90 degree to show the polar body parallel to the oocyte for height gain. PB1 size was calculated by using the formula of 4/3×π×a×b×c (a, b, c represents half of the length, width and height, respectively).

### Statistical analysis

Each experiment was performed at least three times. Statistical analyses of real-time quantitative PCR were conducted using an analysis of variance (ANOVA). Differences between treated groups were evaluated with Duncan's multiple comparison tests. All percentages from more than three repeated experiments were expressed as mean ± SEM. MII plate thickness was expressed as mean ± SEM (µm). Statistical analyses were conducted using Student's t-Test, and a difference of P<0.05 was considered significant.

## Results

### Expression and localization of CK1a during mouse oocyte meiotic maturation and early embryo development

**Figure 1:**
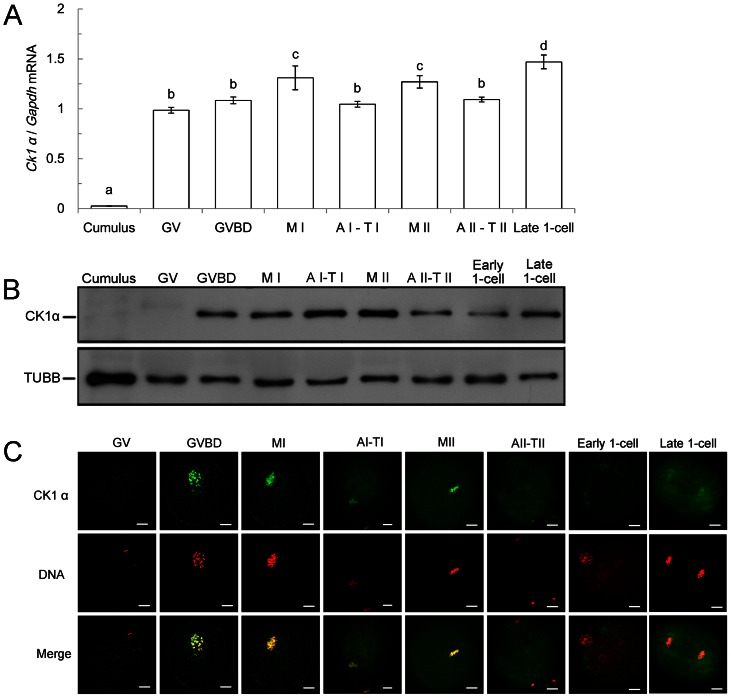
Expression and subcellular localization of CK1α during mouse oocyte meiotic maturation and early embryonic development. (A) Levels of *Ck1α* mRNA revealed by real time RT-PCR analysis. The transcript level of *Ck1α* from cumulus cells, oocytes of GV, GVBD, MI, AI-TI, MII, AII-TII and embryos of late 1-cell stages (n = 50 for each sample) were detected by real-time PCR. Graphical representation demonstrating pattern of *Ck1α* mRNA levels between eight samples obtained from real-time PCR. *Gapdh* was used as internal control. Oocytes from GV stages were normalized to 1. Each bar represents mean ± SEM (n = 3). Different superscripts on the bar indicate statistical difference (p<0.05). (B) Expression of CK1α was detected by western blot. Cumulus cells from 5 mice and 200 oocytes for each sample were lysed in Laemmli buffer. TUBB was used as a loading control. The molecular mass of CK1α and β-tubulin were about 39 kDa and 53 kDa, respectively. The results of one representative of three independent experiments are presented. (C) Subcellular localization of CK1α during oocyte maturation and early embryo development. Oocytes at different stages were stained with antibody to detect CK1α, and DNA was stained with PI. The slides were examined under a confocal microscope. Green: CK1α; Red: DNA; Merge: overlapping of green and red. Scale bar = 10 µm.

In order to study the role of CK1α, we examined CK1α expression and subcellular localization during oocyte maturation and early embryo development. Oocytes were cultured for 0, 2.5, 8, 9.5, and 12 hours, when most of the oocytes reached the stages of GV, GVBD, MI, AI-TI and MII, respectively. Early embryos were obtained at 2, 6, and 15 hours of insemination, which are the time points embryos arrived at the stages of AII-TII, pronuclear and late 1-cell stage, respectively. As shown in figure 1A, in oocytes, *Ck1α* relative mRNA expression is significantly higher in MI and MII (1.32±0.12 for MI, 1.27±0.06 for MII). However, its expression is relatively low in the stages of GV, GVBD, AI-TI and AII-TII (0.98±0.02 at GV, 1.08±0.03 at GVBD, 1.05±0.02 at AI-TI, 1.09±0.02 at AII-TII) (P<0.05). In the late 1-cell stage after fertilization, the *Ck1α* relative mRNA reached the relatively highest abundance (1.47±0.07, P<0.01). In contrast, *Ck1α* was only negligibly expressed in cumulus cells (0.02±0.003, P<0.01).

We next examined the CK1α protein expression by western blot analysis. Our results showed that CK1α was expressed since GVBD occurred; it gradually increased from GVBD to MII stages. After that, a decrease of CK1α expression was observed at the AII-TII and early 1-cell stage, while it recovered at the late 1-cell stage (Fig. 1B). Consistent with real-time PCR result, CK1α protein could not be detected in cumulus cells.

To investigate the subcellular localization of CK1α during oocyte meiotic maturation and early embryo development, we performed immunofluorescent staining with the oocytes and early embryos at different stages. As shown in figure 1C, there was no specific accumulation of CK1α in GV stage oocytes or early 1-cell stage embryo. However, when the congression of DNA occurred (red), CK1α (green) always colocalized with chromosomes, from GVBD to the late 1-cell stage. This colocalization can be defined by the color of yellow (merge of red and green, which represent DNA and CK1α, respectively). After GVBD, chromosomes condensed in preparation for congression. At the same time, CK1α accumulated and colocalized with the chromosomes. A strong signal of CK1α was detected at the MI and MII stages, in which chromosomes were highly congressed. After separation of homologous chromosomes at AI-TI, CK1α was localized to the separated chromosomes. However, the staining of CK1α at AII-TII was faint. This faint staining was consistant with the results of western blot, which showed weak bands at the AII-TII stage. Taken together, accumulation of CK1α was highly correlated with oocytes meiotic progress, strong staining in meiosis or mitosis, and weak staining in interphase.

### Depletion of CK1α causes chromosome misalignment and oocytes maturation failure

**Figure 2:**
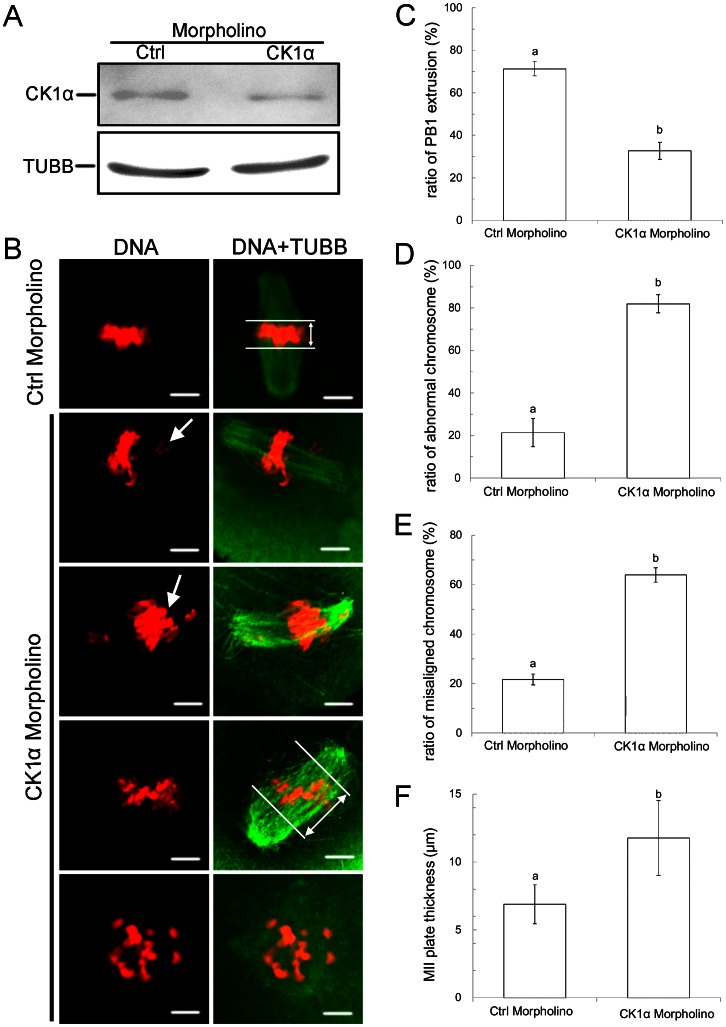
Depletion of CK1α by MO injection caused oocytes maturation failure and chromosome misalignment. (A) Western blot analysis after CK1α MO or control MO injection. Oocytes injected with CK1α MO or control MO were arrested for 20 hrs in milrinone-containing culture medium. Samples were subjected to total protein extraction and western blot analysis. The results of one representative of three independent experiments are presented. (B) Confocal microscopy image of knockdown effect of CK1α MO in oocytes. Oocytes injected with CK1α MO displayed an M II plate with lagging chromosomes (row 2), chromosome segregation failure (row 3), thicker MII plate (row 4), and MII plate with dispersed chromosomes (row 5), whereas control oocytes showed a flat and congressed normal MII plate (row 1). Chromosome lagging and segregation failures are indicated by arrows in row 2 and row 3, respectively. MII plate thickness was measured by the axis distance between two lines at the edges of the red region (PI staining). Green: CK1α; Red: DNA. Scale bar = 5 µm. (C) CK1α MO caused decrease in PB1 extrusion. t-Test was used to determine significance of difference for PB1 extrusion. Different superscripts on the bar indicate statistical difference (p<0.01). (D) CK1α MO caused chromosome abnormalities. At least 25 oocytes were evaluated for each repeat. t-Test was used to determine significance of difference for abnormal chromosome occurrence. Different superscripts on the bar indicate statistical difference (p<0.01). (E) CK1α MO caused misaligned chromosomes. At least 30 oocytes were evaluated for each repeat. t-Test was used to determine significance of difference for misaligned chromosome occurrence. Different superscripts on the bar indicate statistical difference (p<0.01). (F) CK1α MO caused thicker MII plate. At least 25 oocytes were evaluated for CK1α MO or control MO group. t-Test was used to determine significance of difference for MII plate thickness. Different superscripts on the bar indicate statistical difference (p<0.01).

To further explore the functional role of CK1α during oocyte meiotic maturation, we knocked down CK1α by injecting its specific morpholino (MO) into GV-stage oocytes (Fig. 2). Western blot showed that the expression level of CK1α was distinctly reduced (Fig. 2A), which revealed the efficiency of CK1α depletion by MO injection. Since the CK1α was colocalized with chromosomes, the distribution of chromosomes was evaluated after CK1α depletion. In the control group, a normal MII plate with congressed chromosomes was observed (Fig. 2B, row 1). But in the CK1α MO injection group, the oocytes exhibited various kinds of chromosome congression defects (Fig. 2B). We classified chromosome defects into four types. The first type displayed an MII plate with lagging chromosomes. In this type, most chromosomes were congressed at the mid-plate whereas several of them were delayed for congression (Fig. 2B, row 2, arrow). The second type was chromosome segregation failure. In this type, chromosomes can not be pulled apart by spindle for separation (Fig. 2B, row 3, arrow). The third type showed a thicker MII plate with a larger red area (DNA staining) that was perpendicular to the spindle axis (Fig. 2B, row 4, right). The last type displayed the MII oocytes with dispersed chromosomes. In this type, no congression of chromosomes was observed (Fig. 2B, row 5).

The effect of CK1α MO on oocyte PB1 extrusion was determined. As shown in figure 2C, the rate of PB1 extrusion in the CK1α MO injection group was significantly lower than that in the control group (Control MO: 71.3±3.3%, n = 354; CK1α MO: 32.7±3.9%, n = 253, P<0.01). Furthermore, we noted that there was a large proportion of abnormal chromosomes in the CK1α MO injection group compared to the controls (Control MO: 21.7±2.2%, n = 78; CK1α MO: 81.9±4.2%, n = 75, P<0.01) (Fig. 2D). Also, the ratio of misaligned chromosomes in the CK1α MO injection group was much higher than that in the control group (Control MO: 21.4±6.7%, n = 78; CK1α MO: 63.9±2.9%, n = 75, P<0.01) (Fig. 2E). Furthermore, in the CK1α MO injection group, the MII plate was much thicker than that of the control group (Control MO: 6.88±1.43 µm; CK1α MO: 11.77±2.76 µm, P<0.01) (Fig. 2F). All these results indicate that blocking of CK1α with MO severely affects PB1 extrusion and chromosome congression.

### D4476, an inhibitor of CK1, causes oocyte maturation and embryo developmental failure

**Figure 3:**
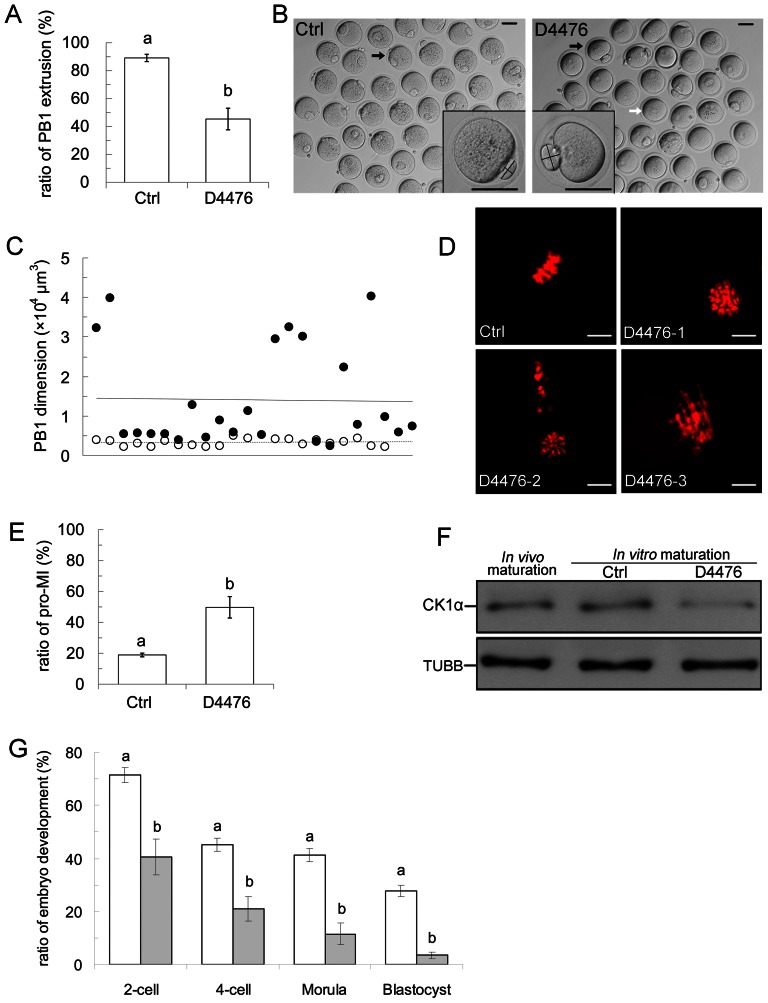
D4476, an inhibitor of CK1, caused oocyte maturation and embryo development failures. (A) D4476 caused a decrease in PB1 extrusion. t-Test was used to determine significance of difference for PB1 extrusion. Different superscripts on the bar indicate statistical difference (p<0.01). (B) Representative images of oocytes incubated in D4476-containing medium and drug-free medium for 12 hours. Most of the oocytes in the control group reached the MII stage. Black arrow indicates representative oocyte with normal PB1 (left). Most of the oocytes incubated with D4476 can not extrude PB1. White arrow indicates representative oocyte without PB1 (right). Some oocytes extruded giant PB1. Black arrow indicates representative oocyte with giant PB1 (right). The representative oocytes are shown enlarged in the corner. Scale bar = 50 µm. (C) PB1 dimension was measured in the control and D4476 treated groups. Hollow dots indicate the dimension of the control group and solid dots indicate the D4476 treatment group. Trendlines of PB1 dimension are shown in solid line (D4476) or dashed line (control). (D) Confocal microscopy image of the effect of D4476 on chromosome congression. Three abnormal types of chromosome conngression are shown: pro-MI stage (D4476-1), dispersive chromosomes (D4476-2), and disorganized chromosome (D4476-3). Red: DNA, Scale bar = 5 µm. (E) D4476 induced a high ratio of pro-MI arrest. t-Test was used to determine significance of difference for the ratio of pro-MI arrest. Different superscripts on the bar indicate statistical difference (p<0.01). (F) Western blot of CK1α in *in vivo* maturation, *in vitro* maturation and D4476-treated oocytes. 200 oocytes for each sample were lysed in Laemmli buffer. TUBB was used as a loading control. The molecular mass of CK1α and β-tubulin were about 39 kDa and 53 kDa, respectively. The results of one representative of three independent experiments are presented. (G) D4476 affected early embryo development. Oocytes were incubated in D4476-containing or D4476-free CZB medium for 12 hours. Oocytes with PB1 were inseminated and embryo developmental rates were recorded. t-Test was used to determine significance of difference between control and D4476 groups. Within the same stage, percentages without a common letter are different (P<0.01).

D4476, a CK1-specific inhibitor, was used as another approach to determine CK1α's functional blocking (Fig. 3). The GV oocytes were incubated in CZB medium containing 50 µmol/L D4476. This concentration is the half maximal effective concentration (EC50) in our preliminary concentration testing experiments (data not shown). After 12 hours of incubation, oocytes were cultured in the D4476-free medium. As shown in figure 3A, the rate of PB1 extrusion in the D4476 group (45.4±7.7%, n = 102) was significantly lower than that of the control group (89.1±2.6%, n = 144) (p<0.01). By contrast, most of the oocytes in the control group extruded normal PB1 (Fig. 3B, left). Although a small number of oocytes accomplished PB1 extrusion in the D4476 group, we noted that these oocytes extruded an abnormal giant polar body. After amplification of these oocytes, a significant giant polar body can be observed. One of the representative oocytes both in the control and in the D4476 groups is shown in the corner in figure 3B. At the same time, we measured the dimension of these polar bodies (dark cross lines in amplified oocytes in figure 3B). After performing calculations, we found that the dimension of the PB1 in the D4476 group (solid dots) is on average 3.6 fold larger than that in the control group (hollow dots). The trendline also suggested differences of PB1 dimensions between these two groups (real line represents the trendline of the D4476 group, dashed line represents the trendline of the control group) (Fig. 3C).

We further wanted to know if D4476 affects chromosome distribution during oocyte maturation. Confocal microscopy identified oocytes in the D4476 group arrested at the pro-MI stage showing abnormal chromosome distribution (Fig. 3D). These abnormal chromosomes could be classified into three types. The first type displayed oocytes remaining in the pro-MI stage (Fig. 3D, D4476-1), the second type displayed two parts of dispersive chromosomes (Fig. 3D, D4476-2), and the third type showed disordered chromosomes (Fig. 3D, D4476-3). The number of the oocytes remaining in the pro-MI stage was recorded. As shown in figure 3E, the proportion of pro-MI stage oocytes in the D4476 treated group (49.8±6.9%, n = 68) is significantly higher than that of the control group (18.9±1.2%, n = 78) (P<0.01).

To investigate the underlying mechanism of D4476 on oocyte meiosis, we examined the protein expression level of CK1α after treatment with D4476. Western blot showed that the expression of CK1α declined in oocytes cultured in CZB containing D4476 compared to the control of *in vivo* or *in vitro* matured oocytes without D4476 treatment (Fig. 3F).

Since D4476 affects chromosome congression and oocyte maturation, we proceeded to investigate if embryos generated from D4476 treated oocytes have impaired developmental potential. *In vitro* fertilization (IVF) was used to inseminate *in vitro* matured (IVM) oocytes treated with or without D4476. Medium used for embryo developmental did not contain D4476. The results showed that the ratio of 2-cell, 4-cell, morula as well as blastocysts in the D4476 group was significantly lower than that in the control group (2-cell, 40.5±6.7% *vs* 71.6±2.9%; 4-cell, 20.9±4.6% *vs* 45.2±2.7%; morula, 11.5±4.0% *vs* 41.2±2.5%; blastocyst, 3.5±1.2% *vs* 27.8±2.1%; P<0.01).

### Pyrvinium pamoate (PP) allosteric activation of CK1α leading to oocyte maturation failure

**Figure 4:**
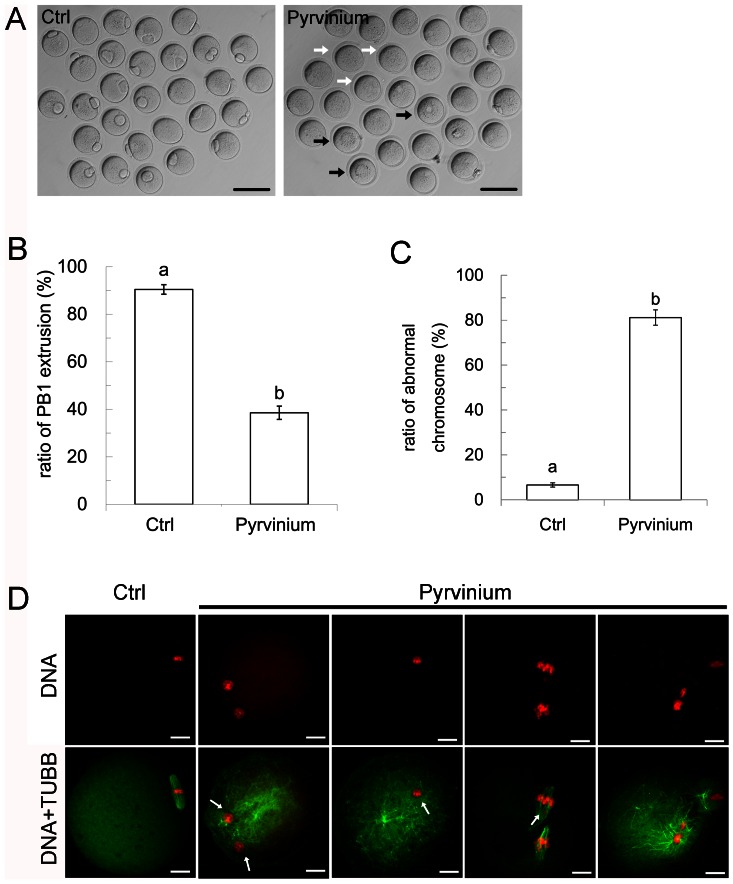
Pyrvinium pamoate allosteric activated CK1α leading to oocyte maturation fail. (A) Representative images of oocytes incubated in PP-containing medium and drug-free medium for 12 hours. Most of the oocytes in the control group reached the MII stage (left), whereas most of the oocytes in the PP-containing medium did not extrude PB1 (right). White arrow indicates representative oocyte without PB1. Black arrow indicates representative oocyte with a dark circle in the cytoplast. (B) PP causes decrease in PB1 extrusion. t-Test was used to determine significance of difference for PB1 extrusion. Different superscripts on the bar indicate statistical difference (p<0.01). (C) PP caused chromosome abnormalities. At least 30 oocytes were evaluated for each repeat. t-Test was used to determine the significance of difference for abnormal chromosome occurrence. Different superscripts on the bar indicate statistical difference (p<0.01). (D) Confocal microscopy image of PP caused chromosome abnormalities. Column 1 showed normal spindle and chromosome congression in the control group. Column 2 and 3 presents abnormally congressed chromosomes (arrow) and spindle assembly failure. Column 4 presents lagging chromosomes (arrow). Column 4 presents oocyte with extruded PB1 but chromosome disorders and failed spindle assembly. Green: β-tubulin. Red: DNA. Scale bar = 10 µm.

Previous reports showed that pyrvinium pamoate (PP) activates CK1α through modifying its conformation [22]. In our preliminary study, GV stage oocytes were incubated in CZB medium containing different concentration of PP from 1.0 nmol/L to 10.0 nmol/L. The concentration of 3.0 nmol/L was the EC50 (data not shown), which was used in the following experiments. Some oocytes were cultured in drug-free medium as control. In the control group, most of the oocytes extruded a normal PB1 (Fig. 4A, left). In the PP group, there were two types of oocyte without PB1 extrusion. One of them contained uniform cytoplasm (Fig. 4A, right, white arrow), the other type had a dark circle in the cytoplasm (Fig. 4A, right, bold arrow), similar to treatment with D4476 (Fig. 3B, right). The ratio of PB1 extrusion was compared between PP and control groups. As shown in figure 4B, oocytes extruding PB1 in the control group had higher nuclear maturation rates (90.4±2.0%), whereas in the PP group most oocytes did not extrude PB1 (38.5±2.7%, P<0.01).

In order to explore the mechanism of oocyte maturation failure by PP treatment, we stained DNA and tubulin using immunofluorescence microscopy. We found that oocytes treated with PP displayed a large proportion of abnormal chromosomes. The ratio of abnormal chromosomes in the PP group is 81.3±3.4%, while that in the control group is 6.6±0.9% (P<0.01) (Fig. 4C,). The morphology of abnormal chromosomes was observed using confocal microscopy. As shown in figure 4D, oocytes without PP treatment displayed normally congressed chromosomes and normal spindles (column 1). However, oocytes treated with PP exhibited different abnormal chromosome types. The first type showed chromosome clustering (Fig. 4D column 2, 3). In column 2 chromosomes present two round parts and spindle assembly failed, microtubules were dispersed in the oocyte. Column 3 is similar to column 2 but chromosomes clustered into a round configuration, and microtubules became distributed in the cytoplasm. The second type showed chromosome lagging (Fig. 4D column 4). In this type chromosome congression failed and homologous chromosomes separated successfully while the PB1 did not extrude. The third type showed oocytes that had a PB1 but chromosomes were disordered and spindle assembly failed (Fig. 4D column 5). All these results suggest that PP mediated over-activation of CK1α impairing PB1 extrusion and chromosome congression during oocyte maturation.

## Discussion

In this study, we examined the CK1α expression profile during mouse oocyte maturation and early embryo development. We further used CK1α morpholino as well as CK1's inhibitor and activator to analyze the relationship between CK1α and chromosome congression/separation. Our results indicate that CK1α plays an essential role in regulating chromosome congression and separation during mouse oocyte meiotic maturation and early embryo development.

Casein kinase 1α has been considered to play a role in chromosome segregation in mitosis, indicating its role as a cell cycle regulator in human and yeast [18]. Previous studies showed CK1α present in mouse oocytes and embryos; injection of CK1α antibody had no effect on second meiosis resumption, but regulated the procession of interphase to mitosis during the first cell cycle [19]. Our previous study showed a CK1α relation to condensed chromosomes, and notable absence in somatic cell nuclear transfer (SCNT) constructs [20]. All these previous studies illustrate that CK1α is correlated with chromosome dynamics in mitosis. Regarding the role of CK1α on meiosis maturation of mouse oocytes, only little information is available.

We detected *CK1α* mRNA, protein expression and subcellular localization during mouse meiosis maturation and early embryo development. Our results clearly showed that *Ck1α* mRNA was expressed from GV to the late 1-cell stage, achieved the highest level in M phase, including MI, MII and metaphase of the 1-cell stage when chromosomes are highly congressed. This co-ocurrence of the peak of *Ck1α* mRNA and chromosome congression implied a role for CK1α in chromosome dynamics. We further confirmed the CK1α expression profile by western blot analysis; CK1α peptide was detected from GVBD and strikingly increased at MI, AI-TI, MII and metaphase of the 1-cell stage. This was consistent with the profile of mRNA levels except for GV. We suppose that in the GV stage oocytes, maternal mRNA of CK1α already existed. However, those mRNA will not translate into protein until an initial signal is produced by the stimulation of germinal vesicle breakdown. On the other hand, gene translation from mRNA to protein takes about two hours in oocytes. This is the result of post-transcriptional regulation. Nevertheless, our results clearly showed the correlation of chromosome congression and Ck1α expression. These results indicate that CK1α plays an important role in the process of oocyte maturation, especially in the process of chromosome congression.

To further study the role of Ck1α during chromosome congression, immunofluorescence and confocal scanning microscopy were employed to clarify the subcellular localization of CK1α in oocytes. Our current study, together with our previous results in MII oocytes and SCNT embyos [20], showed that CK1α is colocalized with condensed and congressed chromosomes. These results conflict with previous reports which showed that CK1α was mainly localized in the spindle area [18]. In this study, CK1α was first reported to localize to vesicular cytolic structures and centrosome in interphase cells, and then associated with spindles in mitosis [18]. Subsequent research found CK1α localized to the plasma membrane in the mouse GV oocyte and in the spindle area in the MII stage [19]. In our study, CK1α-specific staining arose from GVBD, and was associated with chromosomes in meiosis or mitosis. We did not detect any signal in the spindle area. The different results regarding immunofluorescent staining are most probably explained by the different recognition of a related antigen. However, CK1α was localized in chromosomes, suggesting that it may be involved in the regulation of chromosome congression and separation. Previous studies demonstrated that CK1δ/ε phosphorylates Rec8 all along at the chromosome arms and centromeres, subsequently recruiting PP2A/Sgo1 to dephosphorylate centromeric Rec8. Then separase cleaves phosphorylated Rec8 to initiate anaphase I [23]. This model adequately implies that the CK1 family was associated with chromosome dynamics, further clarifying the possibility of our subcellular location results. Nevertheless, the localization of CK1α in different cell cycle stages should be further confirmed by different sources of antibodies and cell types.

The other interesting result is the decreased expression of CK1α in the AII-TII stage (but not AI-TI stage), as revealed by western blot and confocal results. Oocyte meiotic maturation is characterized by one round of DNA replication and two rounds of chromosome segregation. During prophase I, the homologs pair and recombine to form chiasmata bivalents with four chromatids. In metaphase I, segments of sister chromatids are tightly associated through multi-subunit cohesin complexes at the arms, and also at centromeres [23]. In this process, cleavage of the meiosis-specific cohesin's α-kleisin subunit Rec8 along chromosome arms, but not at centromeres, is necessary for homolog segregation [24]. Recent studies identified two CK1 isoforms, δ and ε, that are required for Rec8 phosphorylation and then for its efficient removal during meiosis I [16]. In the CK1 family, each member is highly homologous with more than 50% identical kinase domains [13]–[15]. High expression levels of CK1α in AI-TI but not in AII-TII raises the question whether CK1α has similar regulating mechanisms as CK1 δ and ε. This issue needs further investigation.

Because the expression profile of CK1α suggested a potential role in chromosome dynamic changes, we investigated CK1α function by blocking its role through specific-morpholino injection into GV oocytes. Blocking of CK1α function with morpholinos caused severe protein expression changes as well as chromosome misalignment (Fig. 2A and B). Misalignment of chromosomes indicates that the connection of microtubules with the chromosome centromeres was disturbed at the equatorial plate. Depletion of CK1α may result in the observed defects in the interaction of microtubules and centromeres on chromosomes. Previous studies showed that survivin exhibited analogous functions in the process of meiotic maturation [25]. Another casein kinase family member, casein kinase 2 (CK2) has been shown to directly interact with survivin through β-catenin–Tcf/Lef-mediated transcription [26]. Meanwhile, CK1α also participated in the same pathway [22]. Therefore we suppose that CK1α is involved in chromosome alignment through the Wnt signaling pathway. Furthermore, two homolog chromosomes were connected with each other at metaphase I, and cohesion together with Rec8 mediated the linkage between sister chromatid arms in the bivalent chromosomes [27]. In meiosis, phosphorylation of Rec8 occurs along chromosomes; Sgo1-PP2A prevents the centromeric Rec8 dephosphorylation. Once anaphase I is initiated, separase cleaves phosphorylated Rec8 on the chromosome arms, leading to chromosome separation [28]. Blocking CK1α may cut off the phosphorylation of downstream factors and then relevant proteins are unable to integrate with chromosome arms or centromeres. As a result, anaphase I failed to proceed, and chromosome dynamics are affected, as shown in our study.

Moreover, the chromosome-metaphase plate thickness increased when expression of CK1α was knocked down. It revealed the CK1α promoting chromosome congression during meiosis maturation. Furthermore, a higher proportion of MO-injected oocytes did not extrude the first polar bodies, indicating that oocytes were unable to undergo the metaphase-anaphase transition. In the CK1α morpholino injection group, the oocyte's chromosomes did not align and spindle formation failed. These results indicate that CK1α affect homologous chromosome alignment, congression, and segregation. In contrast, previous research showed thatCK1α-synthesized antibody injection did not impact spindle assembly, chromosomal congression and segregation during meiosis. Microinjection of CK1α antibody into MII oocytes after parthenogenetic activation also did not affect chromosome segregation and PB2 formation. One explanation for this controversial result is that the CK1α antibody did not possess a highly efficient inhibitory effect. In our study, CK1α-MO was designed and synthetized. Compared to siRNA, morpholinos are more stable in biological systems [29] and have a much higher affinity for their complementary RNA sequences [30] without interaction to any significant extent with proteins [31]. Efficient inhibitory effect of *Ck1α* mRNA was observed in our study, which demonstrates the significant impact on compromising CK1α function.

D4476 was reported to be the most useful and potent CK1 ATP-competitive inhibitor [32], [33]. We suppose that D4476 inhibits the CK1 function and thus blocks the CK1-dependent signaling pathway, resulting in defective meiosis. One issue need to mention is that D4476 have the inhibitory effects on other CK1 isoforms besides CK1α. A recent research showed that one of the CK1 isoform ε showed very weak expression in mouse oocytes [34]. Also a recent study compared the effects of siRNAs and D4476 in A375 cell line. siRNAs designed according to different kinds of CK1 isoforms were used. The authors found that only siRNA according to CK1α have the same inhibitory effect with D4476. This means that D4476 effects in A375 cell line should be indeed mainly because of CK1α inhibition [35]. A very recent study detected the effect of PP and D4476 in erythrocytes. As stated above, PP is specific for CK1α and does not affect the activity of other CK1 isoforms. In their results, D4476 has the opposite effects to pyrvinium pamoate. They conclude that the inhibitory effect of D4476 is most likely reflecting effects of CK1α [36]. Thus, we believe in our experiments, CK1α is the main target of D4476 in oocytes. However, the effect of D4476 on the other isoforms of CK1 family needs further investigation.

In order to confirm our hypothesis, D4476 was used to investigate the CK1α function during oocyte meiotic maturation. Six obvious abnormalities were observed: (1) chromosome congression failed; (2) oocytes arrested at pro-MI phase; (3) PB1 extrusion was inhibited; (4) giant PB1 extrusion was observed; (5) expression of CK1α was decreased; (6) the ability of embryo development was impaired.

As discussed above, inhibition of CK1α function will disturb chromosome alignment in first meiosis, which results in chromosome congression failure. A large proportion of oocytes arrest at the pro-MI stage, probably because blocking the CK1 function influences the phosphorylation of proteins related to tightly attaching chromosome arms and prevent the onset of the prophase–metaphase transition. Chromosome alignment is the key event for SAC and anaphase-promoting complex (APC) functions [37]. When the alignment of chromosomes at the mid-plate is disturbed, APC can not be activated, thus resulting in failure of PB1 extrusion.

At times, when SAC is disturbed, a few cells still can initiate the metaphase-anaphase transition [38]. However, chromosome movement is associated with actin filament dynamics [39]. Misaligned chromosomes may results in abnormal actin filament dynamics, which in turn affects the spindle position prior to polar body extrusion [40]. Dislocated spindles are responsible for giant polar body extrusion [41]. In our study, most of the oocytes cultured with D4476 can not extrude PB1. Even if some evade SAC surveillance, they extrude a PB1 that is larger than in the control group.

Our western blot analysis showed that D4476 affected the expression level of CK1α. However, the effect of D4476 on CK1α is mainly through protein phosphorylation regulation, but not mRNA translation [32]. In our results, most oocytes treated with D4476 were arrested at the pro-MI stage. Compare to M stage, the protein abundance is much less at pro-MI stage (Fig. 1B). This can explain the inhibitory effect of D4476 on CK1α protein expression.

Successful embryo development is based on high-quality matured oocytes. In our study, few fertilized zygotes generated from D4476-treated oocytes could develop to blastocysts, suggesting that most of the oocytes have abnormal nuclear maturation. This in turn demonstrates the important role of CK1α in oocyte maturation and early embryo development.

Although inhibition of CK1α by morpholino or D4476 resulted in chromosome congression defects, we do not yet know if activation of CK1α promotes chromosome congression. Recent studies showed that PP increased phosphorylation and allosteric activation of CK1α [22] and it has been widely used for erythrocyte and myocardial repair studies [36], [42], [43]. Effect of PP on oocyte maturation has never been reported. In order to confirm our hypothesis stated above, we employed PP to activate CK1α in oocytes. To our surprise, most oocytes treated with PP exhibited over-congressed chromosomes and compact chromosome clusters. Meanwhile, PB1 extrusion was aborted. An interesting issue is that blocking the function of CK1α by application of morpholino or D4476 results in the oocyte meiotic maturation failure, whereas activating the CK1α by PP also negatively affect maturation. This is probably because endogenous CK1α was activated by PP in an inappropriate phase during the cell cycle. Treatment of PP, results in excess activity of CK1α, which leads to the severe congression abnormalities and meiotic maturation failure.

One explanation for our results is that Ck1α affects the function of securin via β-catenin. Activated Ck1α inhibits the function of the Wnt/β-catenin signaling pathway. Phosphorylation of β-catenin by CK1α at Ser45 is the priming reaction for the proteasomal degradation of β-catenin [44]. Previous studies showed that securin is a target of β-catenin transcriptional activation in colorectal adenomas. High expression of β-catenin induced abnormal chromosome separation [45]. Thus, securin as the key factor in oocyte meiosis [46], [47], was disturbed by constitutively activated Ck1α, which induces the failure of PB1 extrusion as well abnormity of chromosome alignment. However, the detailed mechanism underlying PP in chromosome over-congression still needs further investigation.

In conclusion, our data suggest that CK1α has a pivotal role in regulating chromosome congression and separation during oocyte meiotic maturation and early embryo development.
